# Contribution of Character Strengths to Psychology Stress, Sleep Quality, and Subjective Health Status in a Sample of Chinese Nurses

**DOI:** 10.3389/fpsyg.2021.631459

**Published:** 2021-11-01

**Authors:** Shu-e Zhang, Li-bin Yang, Chen-xi Zhao, Yu Shi, Hong-ni Wang, Xin Zhao, Xiao-he Wang, Tao Sun, De-pin Cao

**Affiliations:** ^1^Department of Health Management, School of Health Management, Harbin Medical University, Harbin, China; ^2^Center for Higher Education Research and Teaching Quality Evaluation, Harbin Medical University, Harbin, China; ^3^Department of Health Management, School of Medicine, Hangzhou Normal University, Hangzhou, China

**Keywords:** nurse, character strengths, health outcomes, mediator, cross-sectional study

## Abstract

**Objectives:** The main objectives of this study were to describe the current state of character strengths (CSs) of nurses; explain how they affect stress, sleep quality, and subjective health status; and reveal the mediating role of stress for the subject matter on the association between CSs, sleep quality, and subjective health status.

**Methods:** A cross-sectional online survey was conducted from September to October 2020 in China. A multistage stratified sampling method was used, and 1,221 valid questionnaires across 100 cities in 31 provinces were collected.

**Results:** For the participants in this survey, the three dimensions of CSs ranging from high to low were caring (4.20 ± 0.640), self-control (3.53 ± 0.763), and inquisitiveness (3.37 ± 0.787). There was difference in CSs scores across age (*F* = 8.171, *P* < 0.01), professional categories (*F* = 5.545, *P* < 0.01), and job tenure (*F* = 9.470, *P* < 0.01). The results showed that CSs significantly affected the psychological stress (β = −0.365, *P*< *0.01*), sleep quality (β = 0.312, *P*< *0.01*), and subjective health (β = 0.398, *P*< *0.01*) of nurses. Moreover, psychological stress partially mediated the association between CSs and both types of health outcomes.

**Conclusion:** In China, the CSs of nurses are at high levels. We find that nurses with high-level CSs are likely to experience less psychological stress and exhibit healthy psycho–physiological responses, which contribute to positive health outcomes. Finally, our study argues that strength-based interventions of positive psychology in hospitals should be provided to minimize threats to the physical and psychological health of health professionals, which is a beneficial choice for future hospital reforms in the domain of occupational health management.

## Introduction

A WHO report warns that there is a global shortage of registered nurses (McGrath, 2020). There is also a significant nursing shortage in China owing to the huge population base. Chinese nurses sustain frequent workload and overtime, facing a range of threats, such as alarming stress (Liu and Aungsuroch, 2019), greater fatigue and burnout syndrome (Wang et al., 2019), and high dissatisfaction and risks of exposure to workplace violence (Berland et al., 2008; Proctor et al., 2011; Zhang et al., 2017), all of which result in poor psychological (Zhao et al., 2018b) and physical well-being (Zhang et al., 2017). The long-term and low-level well-being of nurses can result in negative effects, including reduced job engagement (Itzhaki et al., 2018), decreased job performance (Zhang et al., 2018), and increased turnover (Zhao et al., 2018a), as well as increased risk regarding patient safety, owing to nursing errors. Recently, the mental and physical health problems of nurses have been increasing, which is a threat to not only nurses but also their patients, health care organizations, the health care system, and even the thriving of humans in general (Brown and Gunderman, 2006). There is a growing interest worldwide in positive psychology and its emerging applied field of positive organizational behavior to accelerate well-being, flourishing, and health in different settings of employees (Kaplan et al., 2013); however, limited attention has been paid to the medical setting from the positive psychology perspective. Moreover, positive psychology assumes that individuals have inherent capacities for growth, fulfillment, and happiness through the identification and application of character strengths (CSs) (Seligman and Csikszentmihalyi, 2000). Furthermore, the role of CSs is positive with respect to health outcomes, which is an important aspect of the livelihood of individuals. While a previous study shows that personal differences play an important role in the psychological stress and physical health of a sample of nurses (Melanie et al., 2017), little is known about individual sleep quality and health status in different CSs from a positive psychological perspective. Additionally, the existing literature on the effect of CSs on physical health and psychological health is still inconclusive, especially among health professionals.

### Positive Psychology and CSs

Positive psychology (Seligman, 2018) is regarded as an academic field encompassing CSs, and also positive relationships, experiences, and institutions, all of which aim to enhance individual health, flourishing, and well-being (Seligman and Csikszentmihalyi, 2000). To the best of our knowledge, positive psychology aims to identify these psychological strengths to promote a happy life for individuals and create effective interventions for CS to devote themselves to a better life (Seligman et al., 2005). It insists that CS is different from inherited personality while emphasizing the shaping and cultivation of individual CS by intervening external conditions and cultural and social environments (Cloninger, 2005). With the development of positive psychology, especially advantage psychology and positive organizational behavior, numerous studies have attempted to solve the problem in education, medical treatment, and business management contexts, and further promote individual and organizational prosperity through paying special attention to individual CSs (Wagner et al., 2021). An individual with positive emotion and peak experience facilitates the realization of the ultimate goal of internal members in a workplace by identifying, mastering, realizing, and applying CS to promote individual potential rather than filling disadvantages (Littman-Ovadia and Steger, 2010). Moreover, research on CSs asserts that it can promote individual positive development (Petkari and Ortiz-Tallo, 2018) (e.g., well-being and flourishing) and prevent individual psychopathology (Kim et al., 2018) (e.g., depression and anxiety), which implies that CSs present positivity.

Character strength is regarded as an entire cluster of positive traits vital for a better life, manifesting through a pattern of emotions, thoughts, and behaviors (Park and Peterson, 2009). Dr. Martin Seligman and Dr. Neil Mayerson were the first to define CS (Seligman et al., 2005). They created the values in action (VIA) Institute on Character and identified 24 CSs that were divided into six classes of virtues, as follows: wisdom, transcendence, courage, humanity, justice, and temperance (Seligman et al., 2005). Nowadays, the VIA classification of strengths and virtues provides a framework for studying CSs. However, there are differences of understanding in the structural classification in different countries and cultures, including two-, three-, four-, and five-factor models (Duan and Bu, 2017). Considering the Chinese cultural context, the current study adopted a cross-cultural three-factor model of CSs identified by McGrath and Duan et al. based on a theory-driven framework comprising 24 CSs involving caring, inquisitiveness, and self-control (McGrath, 2015; Duan and Bu, 2017). The inquisitiveness component relates to the CS that describes the curiosity and creativity that link the self to the social environment or social relations (Duan et al., 2012; Duan and Bu, 2017), e.g., curiosity, humor, creativity, social intelligence, and zest. The caring component indicates the CS devoted to maintaining agreeable relations with others (Duan et al., 2012; Duan and Bu, 2017), e.g., fairness, leadership, teamwork, authenticity, and kindness, and refers to the positive cognition, emotion, and behavior of individuals in the process of interpersonal communication (Duan et al., 2012; Duan and Bu, 2017). The self-control component denotes the CS that reflects the regulation and adaptation ability to achieve valuable goals (Duan et al., 2012; Duan and Bu, 2017), e.g., regulation, prudence, judgment, perseverance, and the love for learning. Gradually, CS has attracted the wide attention of scientists in a variety of settings. The impact of CS on mental health has been explored in previous studies in normal populations across various countries and cultures (Harzer and Ruch, 2015). Strength-based intervention studies have shown that individual CSs can effectively increase life satisfaction and well-being, and reduce psychological symptoms and ameliorate depression (Park and Peterson, 2009). Nevertheless, only a few studies have explored the relationship between CSs and health outcomes in the nursing setting, e.g., sleep quality and subjective health status.

### CSs and Health Outcomes

The association between individual traits and health has been widely discussed in different settings. Cross-sectional, longitudinal, and experimental studies demonstrated that CSs were associated with numerous benefits related to work, life, and health outcomes (Kobau et al., 2011). There is a significant positive association between personality traits (e.g., Big Five personality, humorous, and optimistic) and different forms of health outcomes, e.g., mental and physical health (Bucher et al., 2019). Previous studies have already confirmed that CSs have a broad range of significant negative associations with psychological stress (Kalyar and Kalyar, 2018) and burnout (Huber et al., 2019), and symptoms of anxiety and depression (Duan et al., 2020), but have a positive correlation with positive emotions (Güsewell and Ruch, 2012); positive experiences, including life (Buschor et al., 2013) or job satisfaction (Heintz and Ruch, 2020); and also various well-being aspects and so on (Buschor et al., 2013). Previous studies provide evidence that high-level CSs have positive effects on decreased psychological symptoms and faster recovery from illness (Peterson et al., 2006). However, limited scientific studies have found that CSs are related to physical well-being in a sample of nurses. Confirmed studies revealed the effects of a single strength from 24 CSs on psychological and physical well-being (Peterson et al., 2006). For example, a person with a high-level CS of happiness has a more active immune system, an individual with high-level CS of humor exhibits more stable blood pressure and a healthier lifestyle, and an individual with a high-level CS of optimism has a lower risk of cardiovascular disease (Peterson et al., 2006). However, CS is not singular, but plural, and it must be tested as a multidimensional construct. It has already been recognized that plural CS could buffer the necessary psychobiological stress responses of individuals under stressful situations through immediate appearance and subsequent decline to protect mental and physical health with an enduring long-term impact (Li et al., 2017). Therefore, based on the theory of Peterson and Seligman and the above-mentioned content, we assume that a nurse with a high level of CSs is significantly related to health outcomes. Considering the professional characteristics of nursing staff, sleep deprivation caused by shift work is closely related to personal health. Therefore, we consider subjective sleep quality and health status as indicators to evaluate the physical health outcomes of the nurses, whereas psychological stress is regarded as another indicator of psychosocial harm (Elo et al., 2003) in the current study.

## AIM

We describe the current state of CS toward nurses and sociodemographic differences in the CSs. We test the associations between CSs and three types of health outcomes, including (a) psychology stress, as an indicator of mental health, and two indicators of physical health, sleep quality, self-evaluated health status, (b) a general appraisal of sleep quality and (c) self-evaluated health status, respectively.

## Materials and Methods

### Subjects and Procedures

With the use of a multistage stratified sampling method, a total of 100 cities and 1,740 nurses were randomly selected. Chinese mainland consists of 32 provinces, which can be divided into five regions by geographical location: eastern, western, southern, northern, and central. In each geographical region, 20 cities were randomly selected. An anonymous online survey was filled out by representative nurses across 100 cities in 31 provinces from September to October 2020 in China. In the second sampling stage, the appointed nurses were selected as the original deliverers at the beginning of the survey. Thereafter, they were fully informed of the purpose and significance of this study, and their positive cooperation was obtained. Subsequently, a web-page link to our self-administered questionnaire was sent by the deliverers to other nurses *via* mobile phones. Meanwhile, the remaining nurses were colleagues invited by the initial participants. We monitored the survey progress every day. Eventually, we checked the accuracy of data and excluded the questionnaire if (1) the age range was below 20 or above 60; (2) the answering time was <150 s (the minimum time in answering the questions was 150 s in the preliminary investigation); (3) logical contradiction between the answers to similar questions. (Sample: “What is your gender? Its response is female and male” “Are you a female nurse? Its response is yes or no”); and (4) failed the quality control questions, such as how carefully you filled out the questionnaire? All data were checked for consistency by two research members. The web page link was sent to 2,100 participants, albeit a total of 1,740 nurses completed the questionnaires successfully. The final sample selection strictly adheres to exclusion criteria for data management and quality control. In total, 1,221 valid questionnaires were selected, with an effective completion rate of 70.13%. The distribution of demographic variables (gender, age, and level of education) of the representative nurses is comparable to the general Chinese registered nurses recorded in China Health Statistics Yearbook. The inclusion criteria were recognized as (1) being a registered nurse, (2) being currently enrolled in nursing, and (3) consent and voluntary participation in our study.

### Measures

This study questionnaire consists of three main parts, i.e., CS, health outcome, and sociodemographics. Demographic variables include gender, age, marital status, monthly salary income, professional categories, education level, job tenure, and work shift.

#### Measurement of CS

A three-dimensional inventory of CSs (TICS) with 15 items, revised by Duan and Bu (2017) was used for measurement. The TICS has demonstrated reasonable utility for medical populations in a Chinese context (Duan and Bu, 2017). Items were rated by nurses on a 5-point scale, ranging from 1 (“very much unlike me”) to 5 (“very much like me”), with a higher score representing a higher degree of each CS. The TICS has an excellent internal consistency with Cronbach's alpha = 0.892, 0.874, 0.881, and 0.914 for inquisitiveness, caring, self-control, and the total scale, respectively, in a sample of Chinese nurses. Exploratory factor analysis was conducted to explore the structure of CSs. Results revealed that the Bartlett spherical test coefficient is 0, representing an obvious level of significance. The KMO coefficient is 0.901, which is >0.900. The Bartlett spherical test illustrates several common factors among the 15 items.

#### Measurement of Psychological Stress, Sleep Quality, and Subjective Health

A single item was adopted to measure the psychological stress of nurses, which was originally devised by Elo AL, Leppanen A (Elo et al., 2003). The item is “stress means a situation in which a person feels tense, restless, nervous or anxious or is unable to sleep at night because his/her mind is troubled all the time. Do you feel this kind of stress these days? (Elo et al., 2003).” The response ranged from not at all (1) to very much (5). Higher scores indicate higher levels of psychological stress. It has been tested to have a good representation of psychological stress in a variety of contexts and cultures (Sun et al., 2017). Past literature has confirmed that a single-item questionnaire has high validity and sensitivity and that it has been used to measure the levels of sleep quality and subjective health of nurses in a Chinese context (Sun and Askay, 2010). Two single items were addressed together to measure the self-reported health outcomes of nurses. Subjective sleep quality is regarded as the evaluation of a nurse of his/her sleep-related characteristics, and whether nurses are satisfied with these qualities (Buysse et al., 1989). Subjective sleep quality was measured by the question “How would you evaluate your most recent night's sleep?” The response ranged from very bad (1) to very good (5). Subjective health status represents the overall assessment of a nurse of his/her health (Svedberg et al., 2001). We consulted the study by Fein and Skinne (Fein and Skinner, 2015), in which the overall subjective health was estimated by a widely used single-item measure (“In general, what do you think of your health?” 5 = excellent, 4 = very good, 3 = good, 2 = fair, 1 = poor). High scores reflect high levels of health status. Previous studies (Li et al., 2005) showed that the self-reported health status measure was consistently associated with medical outcomes and had cost-effectiveness and accessibility.

### Statistical Analysis

The main statistical methods included descriptive statistical analysis, to describe the demographic information of the participants and the status of CS and Pearson correlation, which was tested to estimate the correlations between CS, psychological stress, sleep quality, and subjective health. We relied on the four-step mediated regression approach recommended by Baron and Kenny (1986) to test the associations and mediation effect of CSs and health outcomes. Socio-demographic differences in the three-dimensional inventory of CSs were examined using an independent *t*-test and one-way ANOVA with the least significant difference tests. For all analyses, the level of significance was set at *P* < 0.05 (two-tailed). The previously mentioned analyses were conducted using SPSS 22.0 (IBM, BM SPSS Statistics for Windows).

### Ethical Considerations

The studies involving human participants were reviewed and approved by the Ethics Committee of Harbin Medical University (ECHMU). However, owing to the anonymous survey approach, written informed consent could not be obtained. Verbal informed consent to participate in this study was approved by the Institutional Review Board of Harbin Medical University and obtained from each of the participants on the front page of the questionnaire. Submitting the questionnaire successfully indicated the consent of a nurse to participate in our study.

## Results

### Demographic Information of the Sample

As shown in Table 1, the age of the participants ranged from 20 to 60 years. Female participants comprised 92.79% of the sample. Most (72.23%) participants had bachelor's degree and above. Among the participants, 45.78% were unmarried, ~52.74% were married, and 1.47% were divorced or without a spouse. Among the 1,221 nurses sampled, ~33.01% of them belonged to the primary professional category called nurse, 45.45% belonged to the primary professional category called nurse practitioner, and 22.93% belonged to the medium category named nurse-in-charge. A total of 584 (47.83%) nurses worked <6 years; 75.84% of nurses reported that they had night-shift work. Differences in CSs scores across age (*F* = 8.171, *P* < 0.01), professional categories (*F* = 5.545, *P* < 0.01), and job tenure (*F* = 9.470, *P* < 0.01) were statistically significant. Moreover, female nurses reported higher scores on “caring” (*t* = 7.619, *P* < 0.01) than male nurses. Nurses with high monthly salary income reported higher scores on “caring” (*F* = 2.354, *P* < 0.05) than those with a lower salary, but there was no gender and income difference in the level of inquisitiveness, self-control, and the overall CSs.

**Table 1:** Characteristics of the participants and sociodemographic differences in the three-dimensional inventory of character strengths (*n* = 1,221).

**Characteristic**	***N (%)***	**Inquisitiveness**	**Caring**	**Self- control**	**CSs**
**Age**					
①20–39	706 (57.82)	3.34 ± 0.761	4.19 ± 0.646	3.47 ± 0.762	3.67 ± 0.586
②30–39	415 (33.99)	3.33 ± 0.806	4.19 ± 0.629	3.56 ± 0.742	3.70 ± 0.598
③40–49	83 (6.80)	3.71 ± 0.810	4.32 ± 0.654	3.93 ± 0.690	3.98 ± 0.604
④50+	17 (1.39)	3.55 ± 0.955	4.34 ± 0.537	3.96 ± 0.923	3.95 ± 0.667
*F/t*		*F* = 6.014 *P* <0.05 ① < ③ ② < ③	*F =* 1.241 *P >* 0.05	*F =* 11.454 *P* <0.01 ① < ② < ③ ① < ② < ④	*F =* 8.171 *P* <0.01 ① < ③; ② < ③ ① < ④
**Gender**					
①Male	88 (7.21)	3.21 ± 0.774	3.80 ± 0.619,	3.41 ± 0.671	3.48 ± 0.619
②Female	1,133 (92.79)	3.38 ± 0.787	4.24 ± 0.760	3.54 ± 0.769	3.72 ± 0.593
*F/t*		*T* = 0.214 *P >* 0.05	*t =* 7.619 *P* <0.01	*t =* 1.462 *P>* 0.05	*t =* 0.245 *P>* 0.05
**Education level**					
①Technical secondary school or below	15 (1.23)	3.13 ± 0.893	4.17 ± 0.744	3.60 ± 1.047	3.64 ± 0.737
②College degree	263 (21.54)	3.37 ± 0.861	4.19 ± 0.689	3.54 ± 0.807	3.70 ± 0.659
③Bachelor's degree	883 (72.32)	3.36 ± 0.761	4.20 ± 0.625	3.52 ± 0.747	3.69 ± 0.576
④Master's degree or above	60 (4.91)	3.54 ± 0.794	4.40 ± 0.583	3.73 ± 0.696	3.89 ± 0.575
*F/t*		*F =* 1.477 *P>* 0.05	*F =* 0.735 *P>* 0.05	*F =* 2.655 *P>* 0.05	*F =* 2.162 *P>* 0.05
**Marital status**					
①Unmarried	559 (45.78)	3.33 ± 0.766	4.19 ± 0.641	3.48 ± 0.754	3.66 ± 0.585
②Married	644 (52.74)	3.39 ± 0.799	4.22 ± 0.640	3.58 ± 0.763	3.73 ± 0.604
③Divorced or loss of spouse	18 (1.47)	3.56 ± 0.983	4.34 ± 0.593	3.42 ± 0.945	3.77 ± 0.735
*F/t*		*F =* 1.147 *P >* 0.05	*F =* 1.933 *P >* 0.05	*F =* 1.536 *P >* 0.05	*F =* 1.795 *P >* 0.05
**Professional categories**					
①Primary: Nurse	345 (28.26)	3.37 ± 0.829	4.15 ± 0.686	3.52 ± 0.787	3.68 ± 0.65
②Primary: Nurse practitioner	555 (45.45)	3.30 ± 0.745	4.20 ± 0.626	3.45 ± 0.742	3.65 ± 0.56
③Medium: Nurse-in-charge	280 (22.93)	3.46 ± 0.793	4.25 ± 0.611	3.67 ± 0.741	3.78 ± 0.58
④High: Associate senior nurse /senior nurse	41 (3.36)	3.59 ± 0.867	4.39 ± 0.585	3.77 ± 0.815	3.92 ± 0.64
*F/t*		*F =* 3.653 *P* <0.01 ② < ③ ② < ④	*F =* 2.553 *P* <0.05 ① < ③ ① < ④	*F =* 6.605 *P* <0.01 ① < ③; ② < ③ ① < ④; ② < ④	*F =* 5.545 *P* <0.01 ① < ③; ② < ③ ① < ④;② < ④
**Job tenure (years)**					
①0–3	403 (33.01)	3.35 ± 0.783	4.17 ± 0.634	3.49 ± 0.746	3.67 ± 0.588
②4–5	181 (14.82)	3.35 ± 0.842	4.17 ± 0.691	3.50 ± 0.826	3.67 ± 0.658
③6–10	342 (28.01)	3.25 ± 0.712	4.17 ± 0.628	3.44 ± 0.736	3.62 ± 0.548
④11–20	219 (17.94)	3.47 ± 0.788	4.23 ± 0.617	3.64 ± 0.733	3.80 ± 0.582
⑤21+	76 (6.22)	3.74 ± 0.870	4.40 ± 0.624	3.96 ± 0.737	4.03 ± 0.628
*F/t*		*F =* 7.291 *P* <0.01 ① < ⑤; ② < ⑤ ③ < ⑤; ④ < ⑤	*F =* 3.105 *P* <0.01 ① < ⑤; ② < ⑤ ③ < ⑤;	*F =* 9.075 *P* <0.01 ① < ⑤; ② < ⑤ ③ < ⑤;	*F =* 9.470 *P* <0.01 ① < ⑤; ② < ⑤ ③ < ④ < ⑤
**Monthly salary income (RMB)**					
①3,000 or below	92 (7.53)	3.21 ± 0.769	4.06 ± 0.687	3.47 ± 0.857	3.58 ± 0.637
②3,001–5,000	240 (19.66)	3.32 ± 0.853	4.16 ± 0.704	3.59 ± 0.778	3.68 ± 0.646
③5,001–7,000	311 (25.47)	3.34 ± 0.771	4.19 ± 0.634	3.55 ± 0.749	3.70 ± 0.581
④7,001–9,000	330 (27.03)	3.43 ± 0.771	4.26 ± 0.602	3.55 ± 0.773	3.74 ± 0.592
⑤9,001 or above	248 (20.31)	3.43 ± 0.764	4.24 ± 0.605	3.47 ± 0.711	3.72 ± 0.560
*F/t*		*F =* 1.959 *P >* 0.05	*F =* 2.354 *P* <0.05 ① < ④; ① < ⑤	*F =* 0.984 *P >* 0.05	*F =* 1.356 *P >* 0.05

### Status of Different Styles of CS Toward Nurses

The means and SDs of the 15-item TICS among Chinese nurses are shown in Table 2. Their average score for 15 categories is higher than the median value. For nurses, the level of three-dimensional CS ranging from high to low was caring (4.20 ± 0.640), self-control (3.53 ± 0.763), and inquisitiveness (3.37 ± 0.787). Pearson's correlation coefficients of the continuous variables are presented in Table 3. All of the variables were significantly correlated with each other (*P*< *0.01*), which indicated that each variable could be used in the subsequent multiple linear hierarchical regression analyses.

**Table 2:** Means (M) and SDs of the 15-item three-dimensional inventory of CS (*n* = 1,221).

**Inquisitiveness**	**M**	**SD**	**Caring**	**M**	**SD**	**Self- control**	**M**	**SD**
Curiosity	3.48	0.917	Fairness	4.11	0.828	Regulation	3.50	0.950
Humor	3.24	1.009	Leadership	4.19	0.814	Prudence	3.51	0.945
Creativity	3.47	0.863	Teamwork	4.19	0.785	Judgment	3.46	0.951
Social intelligence	3.24	0.945	Authenticity	4.20	0.760	Perseverance	3.64	0.861
Zest	3.40	0.973	Kindness	4.32	0.731	Love of learning	3.56	0.922
*M i* = 3.37 ± 0.787 < *M c* = 4.20 ± 0.640 > *M s* = 3.53 ± 0.763								

**Table 3:** Means (M), SDs, and correlations of variables (*n* = 1,221).

**Variables**	**M**	**SD**	**1**	**2**	**3**	**4**	**5**	**6**
1. Inquisitiveness	3.37	0.787	1.00					
2. Caring	4.20	0.640	0.499^*^^*^	1.00				
3. Self control	3.53	0.763	0.559^*^^*^	0.444^*^^*^	1.00			
4. Psychological Stress	2.96	0.963	−0.369^*^^*^	−0.168^*^^*^	−0.222^*^^*^	1.00		
5. Sleep Quality	3.31	0.760	0.388^*^^*^	0.248^*^^*^	0.249^*^^*^	−0.374^*^^*^	1.00	
6. Subjective Health	3.09	0.910	0.323^*^^*^	0.180^*^^*^	0.181^*^^*^	−0.455^*^^*^	0.581^*^^*^	1.00

* *p < 0.05*,

* *p < 0.01*.

### Multiple Linear Hierarchical Regression Models

A series of hierarchical multiple regression analyses were used to test the relationship among different variables, after eliminating the interference of the demographic variables involving age, gender, marital status, education, service years, and professional categories. Our study showed that the CSs of Chinese nurses had a significantly negative influence on psychological stress (β= −0.365*, P*< *0.01*), whereas positive influence on sleep quality (β = 0.312*, P*< *0.01*) and subjective health (β = 0.398*, P*< *0.01*). Moreover, according to the suggestion of Baron and Kenny (1986), psychological stress played a mediating role between CS and sleep quality (β = −0.389*, P*< *0.01*) as well as subjective health (β = −0.268*, P*< *0.01*), as seen in Table 4.

**Table 4:** Multiple hierarchical linear regression models of variables [character strengths (CS), psychological stress, sleep quality, subjective health] (*n* = 1,221).

**Variables**	**Psychological stress**		**Sleep quality**				**Subjective health**			
	***M*1(β)**	***M*2(β)**	***M*3(β)**	***M*4(β)**	***M*5(β)**	***M*6(β)**	***M*7(β)**	***M*8(β)**	***M*9(β)**	***M*10(β)**
**Control variables**										
Age	−0.008	0.023	0.129	0.112	0.125	0.120	0.103	0.080	0.100	0.086
Gender	−0.018	−0.034	−0.037	−0.016	−0.045	−0.029	−0.029	0.001	−0.036	−0.008
Marital status	−0.026	−0.031	0.027	0.032	0.015	0.020	0.006	0.015	−0.003	0.006
Education	−0.021	−0.009	0.099	0.088	0.090	0.085	0.014	0.001	0.006	−0.002
Service years	−0.015	0.008	−0.145	−0.168	−0.152	−0.165	−0.131	−0.160	−0.136	−0.158
Professional categories	0.066	0.045	−0.049	−0.035	−0.019	−0.017	0.019	0.035	0.044	0.047
**Mediating variable**										
Psychological stress					−0.454^*^^*^	−0.389^*^^*^			−0.375^*^^*^	−0.268^*^^*^
**Independent variable**										
Character strengths–Inquisitiveness		−0.365^*^^*^		0.312^*^^*^		0.170^*^^*^		0.398^*^^*^		0.248^*^^*^
Character strengths–Caring		0.020		0.031		0.039		0.073^*^		0.077^*^
Character strengths–Self control		−0.035		−0.006		−0.020		0.030		0.021
*F*	0.441	22.208^*^^*^	3.310	18.400^*^^*^	49.432^*^^*^	40.357^*^^*^	1.202	26.049^*^^*^	29.749^*^^*^	34.870^*^^*^
**R*^2^*	0.002	0.142^*^^*^	0.016	0.114^*^^*^	0.217^*^^*^	0.248^*^^*^	0.006	0.162^*^^*^	0.142^*^^*^	0.217^*^^*^
*Δ*R*^2^*	0.002	0.139^*^^*^	0.016	0.104^*^^*^	0.206^*^^*^	0.028^*^^*^	0.006	0.156^*^^*^	0.141^*^^*^	0.177^*^^*^

*M_1_, M_3_, M_7_: the influence of demographic variables on the psychological stress, sleep quality and subjective health; M_2_, M_4_, M_8:_ the influence of character strengths on the psychological stress, sleep quality, and subjective health; M_5_, M_9:_ the influence of the psychological stress on sleep quality and subjective health; M_6_, M_10:_ the influence of CSs and psychological stress on sleep quality and subjective health*.

* *p < 0.05*,

** *p < 0.01*.

## Discussions

### Status of CSs Among Hospital Nurses in China

**Figure 1:**
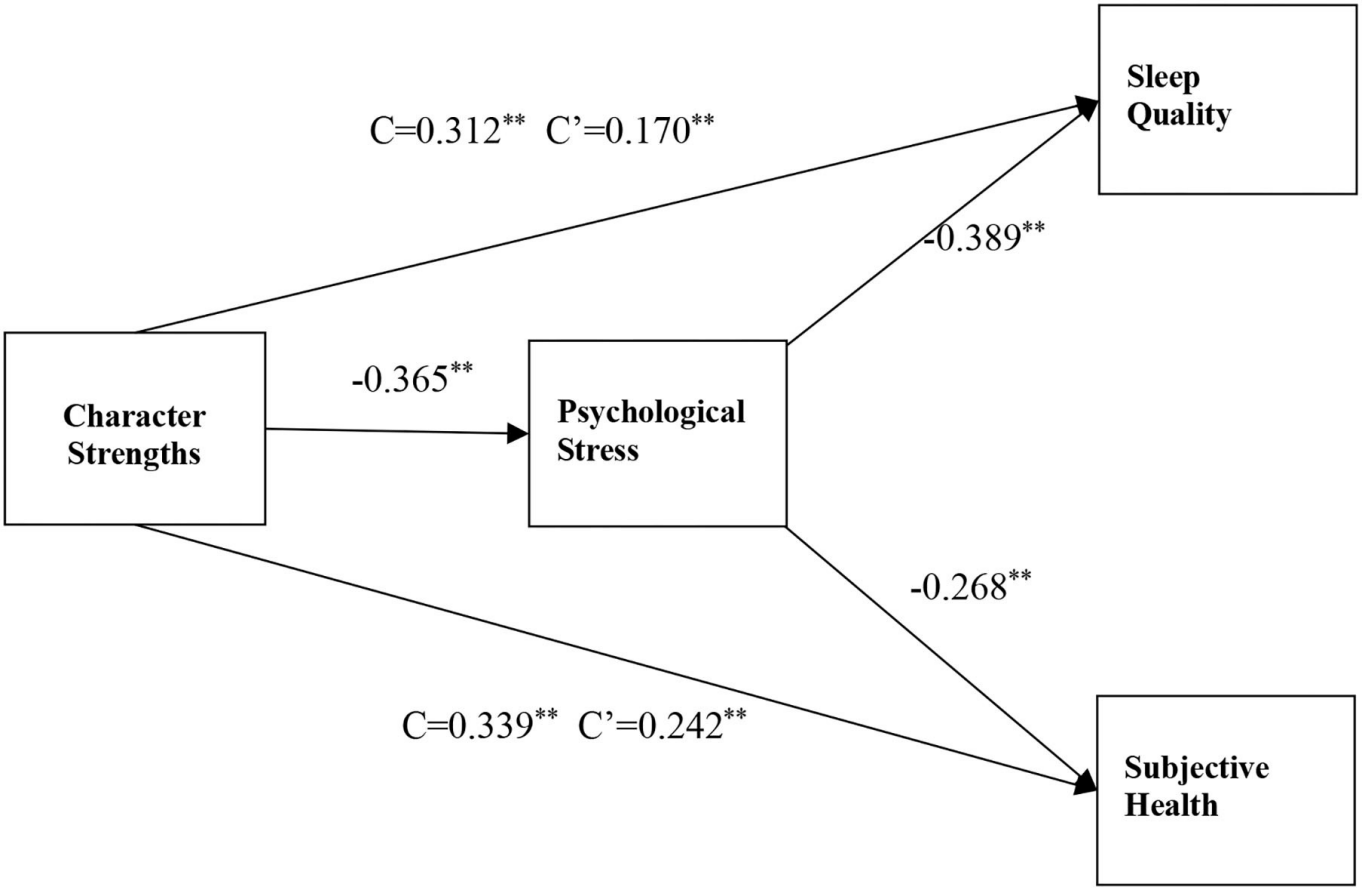
Modified model and standardized path coefficients. ^*^p < 0.05, ^*^^*^p < 0.01.

We used a simple and quick way to assess three-key CSs among nurses. We supported the view that Chinese nurses displayed a relatively high-level three-dimensional CS. Their average score for 15 categories was higher than the median value. For nurses, the level of a three-dimensional CS ranging from high to low was caring, self-control, and inquisitiveness. Moreover, compared with college students, medical inpatients, and community populations, the level of CS of Chinese nurses is also congruent with a previous study (Duan and Bu, 2017). Our study revealed that there were differences in CSs among Chinese nurses by socio-demographic factors involving age, professional categories, and job tenure. This investigation showed that individuals with higher job tenure and older age had higher CS than those with a lower tenure and younger age. Compared with primary nurses, nurses-in-charge and associate senior nurses/senior nurses easily had a higher level of CS. Moreover, female nurses reported a higher level of caring than male nurses, but there was no gender difference in the level of inquisitiveness, self-control, and CS. Overall findings speculated that the CS of a nurse can be changed, cultivated, and shaped at different time points in their life and different settings (Park and Peterson, 2009). Components of the present results are also consistent with several different theoretical positions and empirical trends (Duan and Bu, 2017). Our study presents a suggestion for nursing managers that differences in the CS and sociodemographic factors should be considered in assigning nursing tasks (Figure 1).

### CSs Had a Positive Association With Health Outcomes Among Chinese Nurses

Our results showed that CS positively influenced the sleep quality and health status of Chinese nurses both directly and indirectly. Previous studies revealed that different sets of CS had different influences on health outcomes (Park and Peterson, 2009). Our results also confirmed that the inquisitiveness categories (e.g., curiosity, humor, creativity, social intelligence, and zest) of a three-dimensional CS, rather than the others, had a positive effect on the sleep quality and subjective health status of Chinese nurses. A curious person is devoted to pursuing novel, complex, or challenging interactions and positive experiences (Kashdan et al., 2004). A curious nurse could possess positive emotions, which are likely to promote dopamine release and reduce stress hormones release, being propitious to the individual health and sleep quality, and also a corresponding decrease in heart rate, respiratory rate, and blood pressure for a long time (Kashdan et al., 2004). If the sense of humor can influence specific health outcomes, one confirmed reason or key mechanism is the response to the action of laughing (Bennett and Lengacher, 2008). A study supported the view that laughter decreased stress hormones and could buffer the adverse effects of stress hormones on the immune system of an individual (Bennett and Lengacher, 2008). A creative person can return to a high degree of rationality and integrate his/her inner experiences into a wider reality context, which allows him/her to live healthier (Bungay and Vella-Burrows, 2013). Social intelligence is being aware of the motives and feelings of self and others (Duan and Bu, 2017). Another previous study concluded that social intelligence had a positive impact on health outcomes by improving positive health behaviors (Dumitrescu et al., 2007). Zest is a strength involving life with zeal, vitality, enthusiasm, and vigor, and a zestful nurse is often engaged in more physical activities, leading to higher levels of subjective physical health and sleep quality. Inquisitiveness as a form of CS consists of a multidimensional construct, including a sense of curiosity, humor, creativity, social intelligence, and zest from symptom. Therefore, its impact on the health outcomes of nurses should be easily understood using the multivariate approach in hospital settings. The results hint that the cultivation and shaping of the CSof the nurses are valuable in promoting their physical and psychological well-being.

### Psychological Stress Played a Mediating Role in the Association Between CS and Health Outcome

The present study contributes to the understanding of the underlying mechanisms wherein CSs of the nurses are associated with physical well-being. Consistent with previous findings (Xie et al., 2020), the CSs of inquisitiveness were significantly associated with both physical and mental health, such as less experience of job strain, decreased risk of poor health, and reduced periods of sick leave. Our results showed that CSs are psychological stress-defense factors for Chinese nurses. Moreover, psychological stress played a mediating role in the association between CS and sleep quality, as well as subjective health status. Chinese nurses with high-level CS are prone to show less perceived stress and exhibit better physiological habituation to recurrent stress (Harzer and Ruch, 2015), resulting in achieving higher levels of sleep quality and health status. The inquisitiveness character is not a singular aspect, but rather plural, which is a cluster of positive traits involving curiosity, humor, creativity, social intelligence, and zest, jointly promoting the physical health of nurses (Park and Peterson, 2009). Nurses with CSs of high-level inquisitiveness are more motivated to explore the unknown area and rely on theory and knowledge to solve problems in the nursing workplace, which ultimately triggers a cluster of positive emotions involving increased workplace happiness and relaxation, decreasing the less perceived job stress (Xie et al., 2020). Related studies confirmed that individuals with greater CSs exhibited rapid cardiovascular recovery in response to the stress at baseline and stress exposure (Li et al., 2017), thus, contributing to evidence as effective support for this current result. Moreover, nurses with a high-level CS are likely to present energetic status, growing and experiencing more peak experience, thereby undergoing less perceived stress and rapid stress recovery, which contributes to better sleep quality and health status. Additionally, nurses with greater zest or humor of a type of inquisitiveness are prone to produce less neurotic and less stress hormones, focus more on the positive aspects of life (Harzer and Ruch, 2015), and have better coping mechanisms according to a previous study (Harzer and Ruch, 2015), thus, providing an explanation that zest and humor as a part of CS are significant predictors of nurses' sleep quality and health status. Considering the excessive stress and greater challenges faced by staff in the nursing profession, boosting high-level CS of nurses should be explored to promote general health outcomes. Therefore, our findings suggest that strengths-based intervention approaches should be applied to the hospital settings and nursing workplace to relieve their psychological stress and boom their flourishing in physical and psychological aspects.

## Limitations

Although this study makes significant discoveries *vis-à-vis* both CSs and health outcomes in nursing settings, the following limitations must be addressed. First, the data were collected from self-reports of nurses through an online survey, which may have led to response bias because of social desirability or negative effect. Moreover, although we have shown that the sample is representative of Chinese nurses and there are no significant differences across data collection regions, we acknowledge that the effective completion rate is not ideal. Furthermore, a cross-sectional design could not determine the causal relationship between CSs and health outcomes. We used a single-item measurement tool to assess psychological stress, sleep quality, and subject health status in the current study. Although many studies confirm that the reliability and validity of single-item measurement tools had been certified and were widely used in various countries and populations (Oshagbemi, 1999; Riordan et al., 2018), we do not completely deny the potential deviations. Finally, the method ignores using emotion regulation questionnaires, the behavioral inhibition system, or behavioral activation system scales as a validation step, which should be explored in future research. Further research is also needed to test whether the results are available in different cultural contexts.

## Conclusions

There is a discovery regarding a rarely mentioned variable and mechanism in previous literature. Chinese nurses displayed a relatively high level of CS involving caring, self-control, and inquisitiveness. Physical health, including sleep quality and subject health status, was influenced by the CS of inquisitiveness among Chinese nurses. In this process, psychological stress as an indicator of mental health played a partial mediating role in the association between CS and physical health. In addition, it makes a contribution toward understanding the underlying mechanisms in which the CSs of nurses are associated with both sleep quality and subjective health status. It provided evidence for workplace interventions based on CS as a management approach to promote the physical and psychological health of nurses. Therefore, further research on strength-based interventions strategy should be conducted to improve the physical and psychological health of nurses.

## Data Availability Statement

The datasets used and/or analyzed during this study are available from the corresponding author on reasonable request.

## Ethics Statement

The studies involving human participants were reviewed and approved by the Ethics Committee of Harbin Medical University (No. HMUIRB20200902). However, owing to the anonymous survey approach, written informed consent could not be obtained. Verbal informed consent to participates in this study was approved by the Institutional Review Board of Harbin Medical University and obtained from each of the participants on the front page of the questionnaire. Therefore, once a questionnaire was submitted successfully, we believed the consent of the nurse to participate in our study.

## Author Contributions

DC and TS conceived and designed the study. SZ and TS drafted the manuscript. LY, HW, XZ, XW, and YS collected data and controlled quality. CZ revised the manuscript. YS and SZ conducted the data analyses. All authors contributed to publishing the final manuscript and read and approved the final manuscript.

## Funding

The study was supported by the National Natural Science Fund of China (71774045) to TS and funded by the research projects of scientific start-up at Hangzhou Normal University to TS (2019QDL038).

## Conflict of Interest

The authors declare that the research was conducted in the absence of any commercial or financial relationships that could be construed as a potential conflict of interest.

## Publisher's Note

All claims expressed in this article are solely those of the authors and do not necessarily represent those of their affiliated organizations, or those of the publisher, the editors and the reviewers. Any product that may be evaluated in this article, or claim that may be made by its manufacturer, is not guaranteed or endorsed by the publisher.
